# Novel compound mutations in the mitochondrial translation elongation factor (*TSFM*) gene cause severe cardiomyopathy with myocardial fibro-adipose replacement

**DOI:** 10.1038/s41598-019-41483-9

**Published:** 2019-03-25

**Authors:** Elena Perli, Annalinda Pisano, Ruth I. C. Glasgow, Miriam Carbo, Steven A. Hardy, Gavin Falkous, Langping He, Bruna Cerbelli, Maria Gemma Pignataro, Elisabetta Zacara, Federica Re, Paola Lilla Della Monica, Veronica Morea, Penelope E. Bonnen, Robert W. Taylor, Giulia d’Amati, Carla Giordano

**Affiliations:** 1 Department of Radiological, Oncological and Pathological Sciences, Sapienza University of Rome, Policlinico Umberto I, Viale Regina Elena 324, 00161 Rome, Italy; 2 Wellcome Centre for Mitochondrial Research, Institute of Neuroscience, Newcastle University, Newcastle upon Tyne, NE2 4HH UK; 3 Department of Biochemical Sciences “A. Rossi Fanelli”, Sapienza University of Rome, P.le Aldo Moro 5, 00185 Rome, Italy; 4 Cardiomyopathies Unit, Division of Cardiology and Cardiac Arrhythmias, San Camillo-Forlanini Hospital, Rome, Italy; 5 Department of Cardiac Surgery and Transplantation, San Camillo-Forlanini Hospital, Rome, Italy; 6 National Research Council of Italy, Institute of Molecular Biology and Pathology c/o Department of Biochemical Sciences “A. Rossi Fanelli”, Sapienza University of Rome, P.le Aldo Moro 5, 00185 Rome, Italy; 7 Department of Molecular and Human Genetics, Baylor College of Medicine, Houston, TX 77030 USA

## Abstract

Primary mitochondrial dysfunction is an under-appreciated cause of cardiomyopathy, especially when cardiac symptoms are the unique or prevalent manifestation of disease. Here, we report an unusual presentation of mitochondrial cardiomyopathy, with dilated phenotype and pathologic evidence of biventricular fibro-adipose replacement, in a 33-year old woman who underwent cardiac transplant. Whole exome sequencing revealed two novel compound heterozygous variants in the *TSFM* gene, coding for the mitochondrial translation elongation factor EF-Ts. This protein participates in the elongation step of mitochondrial translation by binding and stabilizing the translation elongation factor Tu (EF-Tu). Bioinformatics analysis predicted a destabilization of the EF-Ts variants complex with EF-Tu, in agreement with the dramatic steady-state level reduction of both proteins in the clinically affected myocardium, which demonstrated a combined respiratory chain enzyme deficiency. In patient fibroblasts, the decrease of EF-Ts was paralleled by up-regulation of EF-Tu and induction of genes involved in mitochondrial biogenesis, along with increased expression of respiratory chain subunits and normal oxygen consumption rate. Our report extends the current picture of morphologic phenotypes associated with mitochondrial cardiomyopathies and confirms the heart as a main target of *TSFM* dysfunction. The compensatory response detected in patient fibroblasts might explain the tissue-specific expression of *TSFM*-associated disease.

## Introduction

Primary mitochondrial dysfunction is an under-appreciated cause of cardiomyopathy, especially when cardiac symptoms are the unique or prevalent manifestation of disease. Homoplasmic mutations in mitochondrial (mt)-tRNAs are paradigmatic of the clinical elusiveness of mitochondrial aetiology. Patients develop a cardiomyopathy with hypertrophic phenotype, which is often isolated (i.e. not associated with typical extra-cardiac features of mitochondrial disease) and can rapidly progress to heart failure^1^. The mitochondrial aetiology in these cases often represents an unexpected finding at cardiac transplant or autopsy, as typically described in families bearing the m.4300 A > G variant in the *MTTI* gene^2^. Useful clinical clues for identifying cardiomyopathies with an underlying pathogenic mechanism are maternal inheritance or the presence of other clinical signs, such as mild blood lactate increase, hearing loss, or subclinical myopathy^3^. A histochemical hallmark of mitochondrial cardiomyopathy is the presence of cytochrome *c* oxidase (COX)-deficient cardiomyocytes. Definitive diagnosis requires the demonstration of combined respiratory chain enzyme deficiency on cardiac muscle, which indicates a generalized defect in mitochondrial translation^3^, or a confirmed molecular genetic diagnosis. In recent years, the advent of high-throughput next generation sequencing has expanded the spectrum of genetic alterations that lead to mitochondrial cardiomyopathy (reviewed in^4,5^). In addition to mutations in the mitochondrial genome encoding mt-tRNAs, autosomal recessive pathogenic variants have been reported in nuclear genes encoding key proteins implicated in translation. These include structural mitoribosomal proteins; mt-aminoacyl-tRNA synthetases; mt-tRNA-modifying enzymes; and initiation, elongation and termination factors of translation (see for review^6,7^). These autosomally-driven mitochondrial disorders are usually associated with an early-onset, severe (often fatal) clinical course, with variable phenotypes. Cardiac specific presentations may occur, in the form of infantile hypertrophic cardiomyopathy^8–12^.

Here, we describe a case of mitochondrial cardiomyopathy with a severe dilated phenotype and pathologic features of biventricular fibro-adipose replacement in a 33-year-old woman who underwent cardiac transplant. Whole exome sequencing revealed novel, compound heterozygous variants in *TSFM* [NM_001172697.1; MIM 604723], encoding the mitochondrial translation elongation factor (EF-Ts). A detailed molecular analysis revealed almost complete absence of the protein variant in the affected myocardium as compared to controls, confirming the pathogenic role of the identified mutations. Analysis of mutant fibroblasts suggests possible explanations for the tissue specific expression of the disease.

## Results

### Patients

Patients demographic data are summarized in Supplementary Table S1.

#### Clinical history of the index case

**Figure 1:**
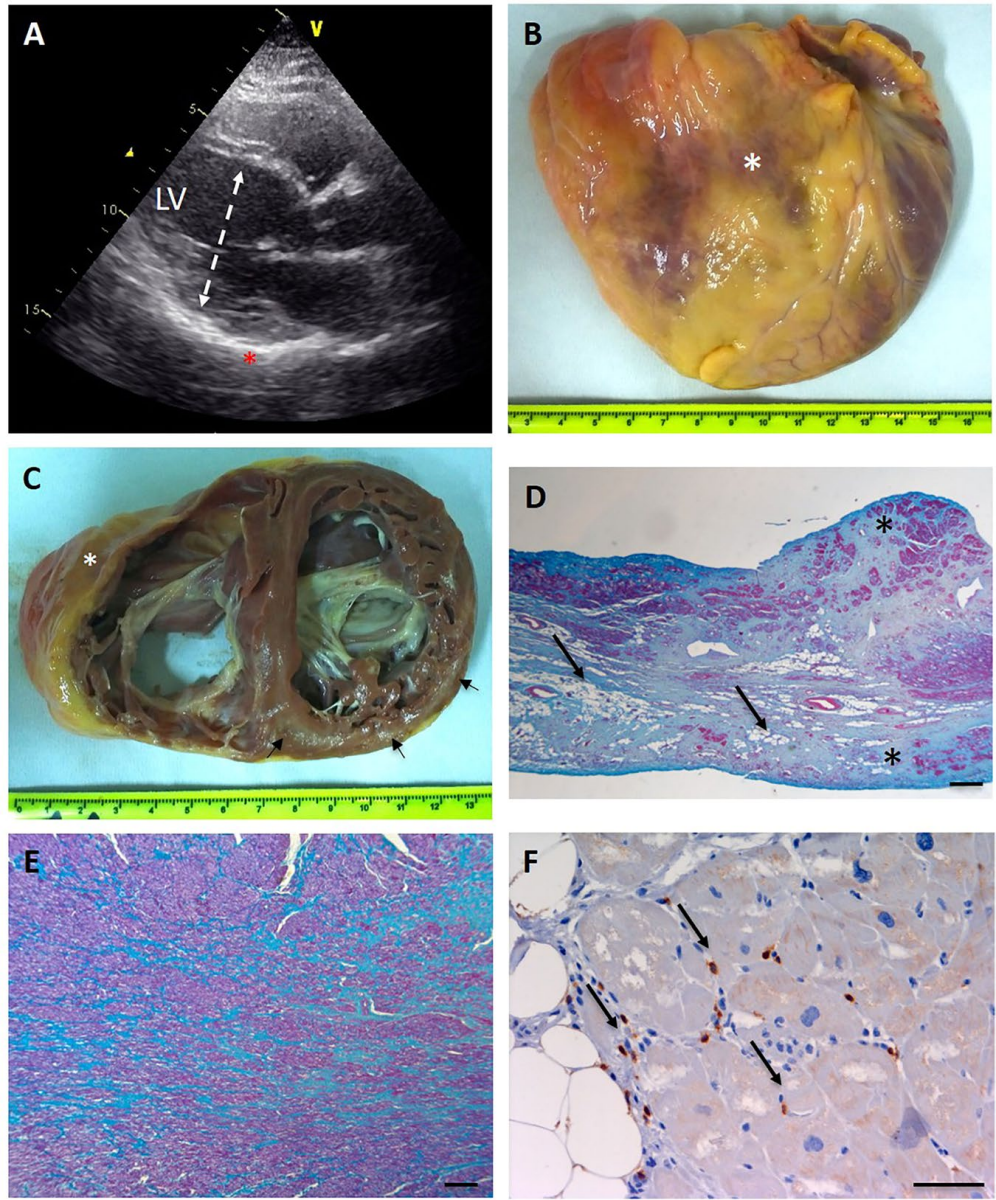
Morphologic features of explanted heart. (**A**) Echocardiographic long axis view at the time of diagnosis, showing a mildly dilated left ventricle (dashed double sided arrow) with an end diastolic diameter (LVDD) of 51 mm. Note the increased wall ecoreflexibility (asterisk). (**B**) Frontal view of the enlarged heart (transverse diameter 120 mm) with a depressed infundibulum consistent with aneurysmal dilation of the anterior wall of the right ventricle (asterisk). (**C**) Cross section of the heart showing biventricular enlargement (RV chamber diameter 50 mm; LV chamber diameter 55 mm). Foci of fibroadipose myocardial replacement are evident in the anterior (asterisk) and posterior right ventricular wall, and the subepicardial and midventricular layers of the left posterior wall (arrows). (**D**) Masson trichrome stain performed on a RV section shows abundant connective tissue (blue) replacing cardiac myocytes (red). Adipose tissue is indicated by arrows. Transmural fibroadipose replacement is indicated by asterisks. (Scale bar 250 μm). (**E**) Foci of scarring (showed in blue) are evident in the LV (Masson trichrome stain, scale bar 250 μm). **(F**) Patchy inflammatory T lymphocytic infiltrate are showed in brown (arrows), (immunohistochemical reaction with anti CD3 antibodies, scale bar 50 μm). RV = right ventricle; LV = left ventricle.

The index case was a 33-year-old female born at term to non-consanguineous healthy parents. She had a younger healthy sister. Pregnancy and delivery were uneventful with normal early psychomotor development. Since early childhood, gross- and fine-motor clumsiness and frequent falls were observed, followed by mild muscle weakness and reduced lower limb coordination. Cognitive function was normal. Genetic screening for Friedreich and spinocerebellar ataxia (SCA) genes had been performed, with negative results. Symptoms remained stable until age 27, when she referred to the Cardiomyopathies Unit of San Camillo-Forlanini Hospital of Rome because of worsening fatigue and dyspnoea. Rest 12-lead electrocardiography showed sinus rhythm in absence of repolarization and depolarization abnormalities. Echocardiography revealed a mildly dilated left ventricle (LV), with increased wall ecoreflexibility (Fig. 1A), LV end-diastolic diameter (LVDD) of 51 mm and ejection fraction (EF) of 21%, consistent with dilated cardiomyopathy. Serum lactate level was slightly increased (3.70 mM; control <2.2 mM). Electromyography, electroneurography and neuro-ophtalmological analysis were normal. Despite medical therapy, LV function progressively decreased. At the age of 33 years, echocardiography showed a hypokinetic and dilated LV, LVDD of 54 mm, ventricular septum thickness of 10 mm, and EF of 15%. The patient underwent cardiac transplant a few months later.

### Controls

Controls consisted of failing hearts (FH, n = 4) obtained from patients who underwent cardiac transplantation because of idiopathic dilated cardiomyopathy (DCM); and non-failing hearts (NFH, n = 4) obtained from donors which were unsuitable for transplantation for technical reasons (see Material and Methods section). All the explanted hearts were analysed at the Cardiovascular Pathology unit of Policlinico Umberto I. Morphological data and representative gross and histological pictures of both FH and NFH are provided in Supplementary Information (Supplementary Table S1 and Supplementary Fig. S1).

### Morphologic analysis and diagnosis of mitochondrial cardiomyopathy

**Figure 2:**
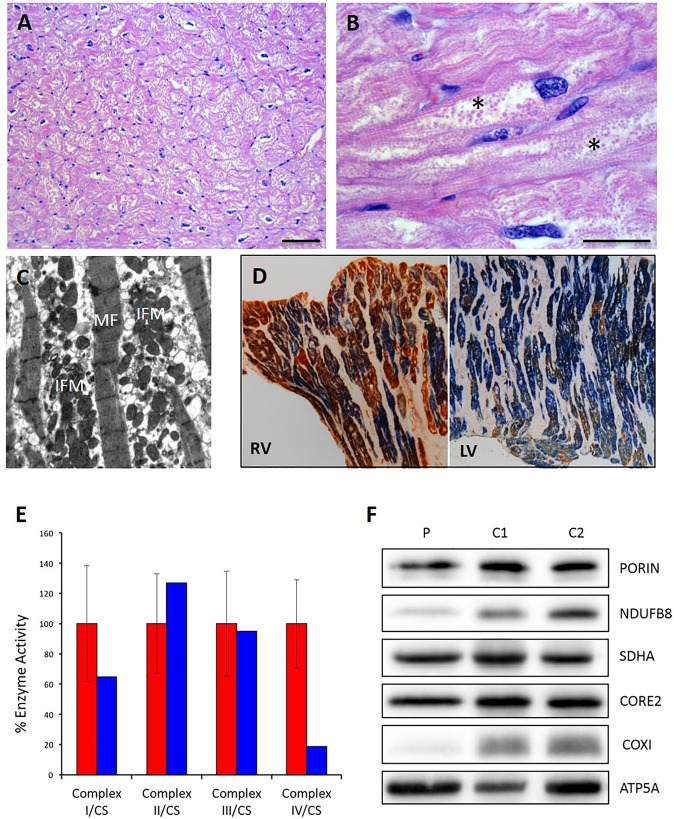
Morphological, biochemical and molecular evaluation of mitochondria on cardiac tissue. (**A**) Hypertrophic cardiac myocytes with vacuolated sarcoplasm in the left ventricle (Hematoxylin and eosin, scale bar 100 μm). (**B**) Vacuoles appeared filled with round-shaped organelles consistent with mitochondrial proliferation (asterisks) (Hematoxylin and eosin, scale bar 50 μm). (**C**) Ultrastructural analysis shows longitudinally arranged cardiac myocytes with clusters of interfibrillar mitochondria (IFM) causing displacement of myofibrils (MF). Mitochondria appear round-shaped and heterogeneous in size (uracyl acetate lead citrate). (**D**) Combined COX/SDH stain shows several isolated COX-deficient cardiomyocytes both in the RV and LV; this feature is more prevalent in the LV. (**E**) Biochemical analysis of mitochondrial respiratory chain enzyme activities performed on cardiac homogenate from the proband and controls. Enzyme activities are normalized to citrate synthase (CS) activity and expressed as percentage of controls (red: controls, blue: patient). (**F**) Western blot analysis of nuclear- and mitochondrial-encoded proteins performed on cardiac homogenate from the proband (P) and controls (C1, C2). RV = right ventricle; LV = left ventricle.

On external examination, the heart from the index case showed a globoid shape, with aneurysmal dilation of the anterior wall of the right ventricle (Fig. 1B). Coronary arteries were unremarkable. On short-axis section, both ventricles were dilated. There were multiple foci of myocardial fibro-adipose replacement, involving the anterior and posterior right ventricular wall and the infundibulum (with occasional transmural extension) and the subepicardial and midventricular layers of the left ventricular posterior wall (Fig. 1C). At histology, myocardial replacement was mostly fibrous in the left ventricle, and mostly adipose in the right ventricle. Patchy CD3+ lymphocytic infiltrates, without myocyte necrosis (Fig. 1D,E) were often detected in the surrounding myocardium (Fig. 1F). Residual cardiac myocytes appeared hypertrophic and showed vacuolated sarcoplasm filled with round-shaped organelles, consistent with mitochondria (Fig. 2A,B). Since fibro-adipose myocardial replacement is a typical feature of arrhythmogenic cardiomyopathy (ACM) due to inherited mutations in desmosomal proteins^13,14^, we looked by immunohistochemistry for reduced expression of plakoglobin at myocytes intercalated disks, which has been reported in ACM^15,16^ but did not detect any difference in the expression level of the protein as compared to controls (Supplementary Fig. S2). On the other hand, ultrastructural analysis detected a marked intermyofibrillar mitochondrial proliferation (Fig. 2C), in agreement with the histologic features of cardiac myocytes, suggesting a mitochondrial aetiology. To verify this hypothesis, the histocytochemical reaction for sequential cytochrome *c* oxidase/succinate dehydrogenase (COX/SDH) was performed, revealing a mosaic pattern of COX-deficient cells, most marked in the left ventricle (Fig. 2D). Accordingly, biochemical analysis of mitochondrial respiratory chain activities performed on cardiac homogenate showed combined deficiency of Complex (C)-I (approximately 60% of the control mean) and C-IV (below 20% of the control mean) (Fig. 2E). This feature was paralleled by reduction of the steady-state levels of selected C-I and C-IV subunits, while C-II, C-III and C-V subunits were spared (Fig. 2F).

### Identification of *TSFM* mutations by whole-exome sequencing

**Figure 3:**
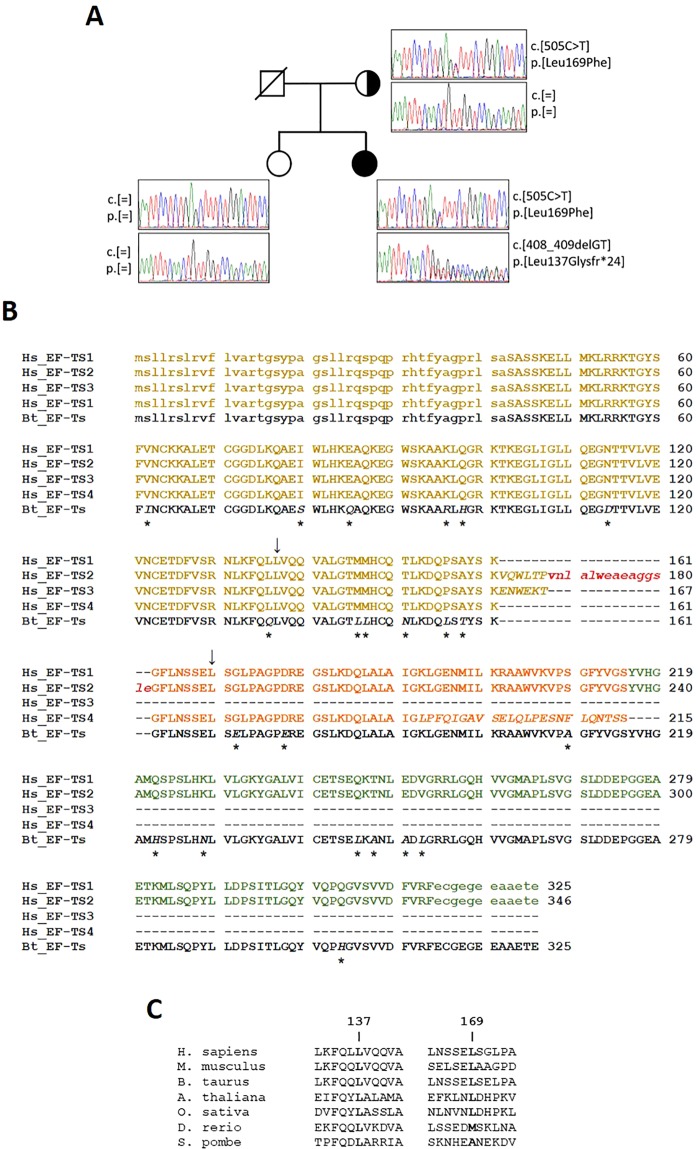
Sequence analysis of *TSFM*. (**A**) Pedigree and chromatograms of the two identified mutations. Both mutations were confirmed to be heterozygous. The black circle indicates the proband, strike-through indicates deceased subject. (**B**) Multiple sequence alignment of the four human mitochondrial EF-Ts protein isoforms and the bovine homologue (Bt_EF-Ts), whose 3D structure is available from the PDB (PDB ID: 1XB2). Upper- and lower-case letters indicate residues present and absent, respectively, in the crystal structure of bovine EF-Ts and in the models of the human isoforms. Residues that differ from isoform 1 are italic and positions where the bovine template differs from isoform 1 are indicated by asterisks. Sites affected by mutations described in this work are indicated by arrows. Sequences are highlighted as follows: yellow, region encompassing the entire isoform 3 (residues 43–167); red, loop region only present in isoform 2 (residues 168–182); orange, isoform 4 region following isoform 3 (residues 162–215, according to isoform 1 numbering); green, isoform 1 and 2 region following isoform 4 (residues 216–325, isoform 1 numbering). **(C)** Conservation of leucine residues at positions 137 and 169 of EF-Ts isoform-1 in a few representative eukaryotic species. The results of an extensive conservation analysis over more than 3,900 species are shown in Supplementary Fig. S3 and discussed in the text.

To identify the genetic variants responsible for the observed cardiac phenotype we first performed sequence analysis of the complete mitochondrial genome, but all of the identified sequence variants corresponded to previously reported polymorphisms described in the MitoMAP compendium (www.mitopat.org, Jun 28^th^ 2018); no pathogenic mtDNA variants were detected (Supplementary Table S2). Quantitation of mtDNA content in cardiac homogenate showed normal mtDNA copy number [mtDNA copy number per cell = 3353 versus 3197 ± 487 in age-matched controls (n = 7)]. Subsequently, whole exome sequencing was undertaken leading to the identification of two novel, heterozygous variants in the *TSFM* gene (NM_001172697.1): one frameshift mutation, c.408_409delGT p.(Leu137Glyfs*24) and a missense variant, c.505C > T p.(Leu169Phe) (Fig. 3A). *TSFM* encodes for a mitochondrial translation elongation factor protein (EF-Ts) that catalyzes the exchange of guanine nucleotides on the translation elongation factor Tu (EF-Tu) during the elongation step of mitochondrial protein translation^17^. Mutations in this gene have been identified as a cause of autosomal recessive infantile-onset mitochondrial cardiomyopathy, encephalopathies with optic and/or peripheral neuropathy, ataxia and Leigh syndrome^18–20^. The c.408_409delGT p.(Leu137Glyfs*24) variant introduces a shift in the translation reading frame of the *TSFM* transcript, resulting in the introduction of a premature termination codon, and is therefore deemed to be pathogenic. The c.505C > T p.(Leu169Phe) variant is not listed on current SNP database (www.ncbi.nlm.nih.gov/SNP/) and has not been previously detected in large-scale sequencing studies (http://evs.gs.washington.edu/EVS/; http://exac.broadinstitute.org/) to date. This variant predicts the exchange of a highly conserved leucine residue by phenylalanine, with this substitution likely to affect the stability of the EF-Ts/EF-Tu complex (see below); additionally, *in silico* analysis (Alamut software v2.7.1) provides additional support in favour of pathogenicity.

Orthogonal validation by Sanger sequencing confirmed the presence of the two variants in the proband. DNA from her father was not available; however, her healthy mother was shown to be heterozygous for p.(Leu169Phe) *TSFM* variant, while the p.(Leu137Glyfs*24) *TSFM* frameshift variant was not observed (Fig. 3A). The healthy sister of the proband did not carry either variant.

### Bioinformatics analysis of the effect of mutations on EF-Ts structure

To evaluate the effects of the observed variants on the EF-Ts protein structure, a detailed bioinformatic analysis was undertaken. According to both the NCBI Gene (www.ncbi.nlm.nih.gov) and UniProt (http://www.uniprot.org/) databases, alternative splicing of the *TSFM* gene encodes four protein isoforms (see Materials and Methods for UniProt and NCBI sequence identifiers). These isoforms are identical to one another in the N-terminal region (residues 1–161) and differ in the C-terminal region (Fig. 3B). Of the two mutations reported above, p.(Leu137Glyfs*24), which is followed by chain truncation, occurs in the conserved N-terminal region and, therefore, has the same sequence numbering in all isoforms. Conversely, the p.(Leu169Phe) mutation occurs in the variable C-terminal region, at position 169 in isoforms 1 and 4, and 190 in isoform 2. Isoform 3 is not affected by this mutation since it is only 167 residues long.

Conservation of the residues at mutated positions was investigated by building a multiple sequence alignment (MSA) comprising EF-Ts protein homologues from over 3900 different species. In this MSA, p.Leu137 is conserved in nearly 48% of the species, whereas Gly is nearly absent at that position; in no species residue 137 is followed by truncation. Leu at position 169 (throughout the text we use the sequence numbering of isoform 1 (having identifiers P43897-1 and NP_005717.3 in the UniProt and NCBI databases, respectively; see Material and Methods) is even more conserved, since it occurs in 60% of the sequences, followed by Ala (15%) and other hydrophobic, neutral or polar residues (i.e., Val, Phe, Ile, Met, Gln, Thr, Ser and Gly) with percentages between 8% and 1% (Fig. 3C and Supplementary Fig. S3).

**Figure 4:**
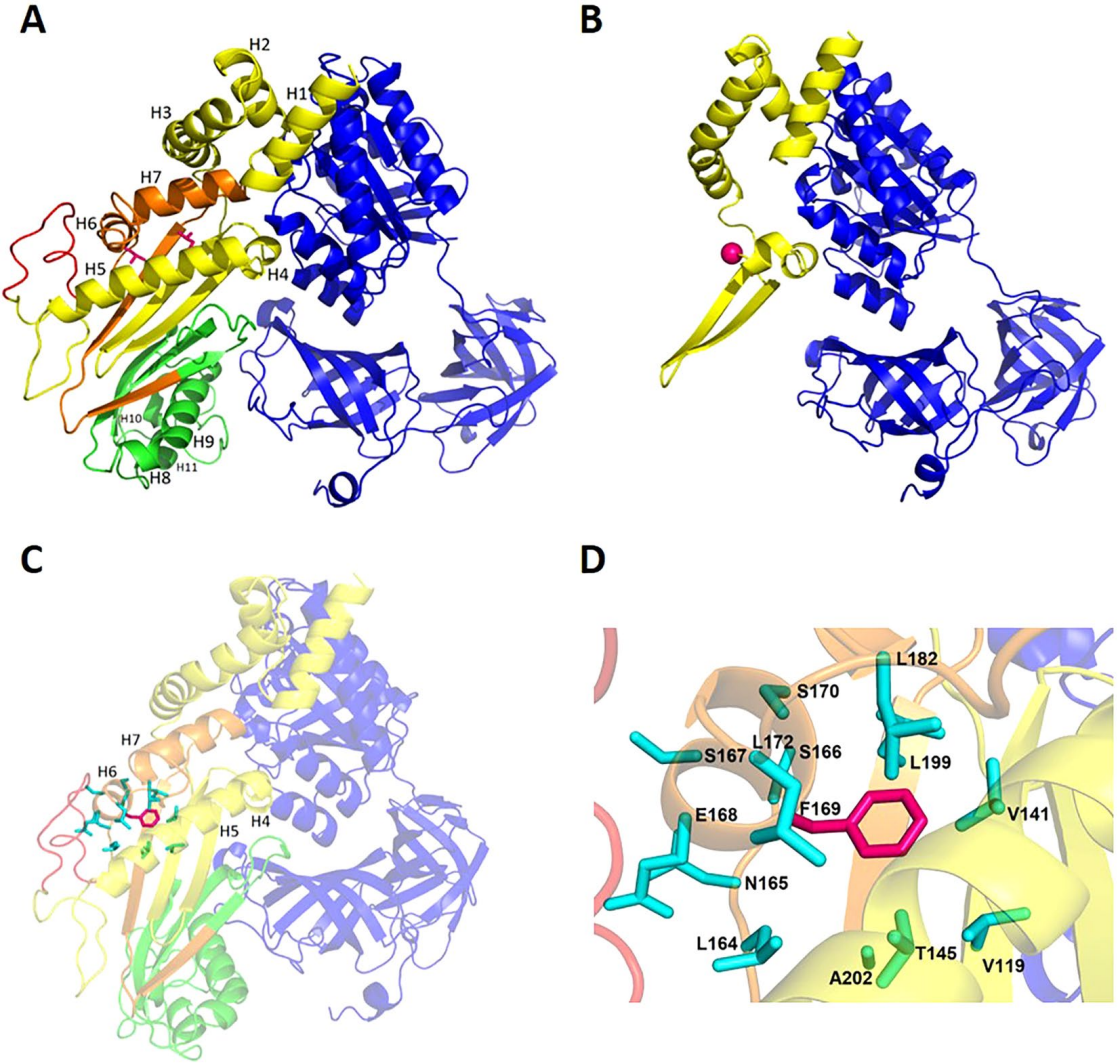
Homology models of EF-Ts isoforms in complex with EF-Tu. (**A**) Mapping of EF-Ts protein isoforms on the homology model of isoform 2 in complex with EF-Tu. The protein main chain atoms are represented as cartoon and colour-coded as follows: blue, EF-Tu; yellow, EF-Ts isoform 3; yellow and orange, EF-Ts isoform 4; yellow, orange and green, EF-Ts isoform 1; yellow, orange, green and red, EF-Ts isoform 2. Leu137 and Leu169 (according to isoform 1 numbering) are shown as sticks and coloured magenta. (**B**) Effect of the Leu137Gly mutation followed by truncation on all isoforms. Gly137 Cα atom is shown as a sphere and coloured magenta. (**C**) Effect of the Leu169Phe mutation on isoforms 1, 2 and 4. The side-chain of Phe169 (magenta) and of surrounding residues (cyan) are shown as sticks; to highlight them, the rest of the protein is made slightly transparent. (**D**) Close view of Phe169 (magenta) and of surrounding residues (cyan), both shown as sticks. Colour-coding is as in panel C.

Building and analysis of homology models of all EF-Ts wild-type and mutant protein isoforms (Fig. 4A–D) allowed the structural role of the mutations and their effect on the interaction with EF-Tu to be investigated. The truncation of all isoforms following p.Leu137Gly mutation is likely to have a dramatic effect on the interaction with EF-Tu. Even if the residual part of the molecule remained folded as in the wild-type (Fig. 4B), the EF-Ts surface involved in interaction with EF-Tu would be greatly reduced (from about 1570 to 1075 Å^2^), resulting in weaker binding. In the wild-type model (Fig. 4A), p.Leu169 is tightly packed with a hydrophobic core comprised of the side-chains of residues Val141, Thr145, Leu164, Leu172, Leu182, Leu199 and Ala202. Replacement of Leu169 with the bulkier Phe in EF-Ts isoforms 1, 2 and 4 determines the establishment of unfavourable van der Waals contacts with surrounding residues (Fig. 4C,D). Release of these clashes is likely to require main-chain, as well as side-chain movements, and to involve a shift in the relative positions of helices H5, H6 and H7, where both Phe169 and residues making clashes with it are located (Fig. 4C). Since helices H5 and H7 are at the interface with EF-Tu, their movement is expected to reverberate on EF-Ts/EF-Tu interaction.

### Marked decrease of EF-Ts and EF-Tu proteins in cardiac tissue from the proband

To confirm whether destabilization of the EF-Ts/EF-Tu complex was responsible for the cardiac phenotype, we analyzed the expression of EF-Ts and EF-Tu proteins and cDNA in myocardial samples from patient and controls. We also evaluated the expression of EF-G1 (*GFM1* gene), a mitochondrial elongation factor that it is not supposed to interact directly with the EF-Ts/EF-Tu complex.

**Figure 5:**
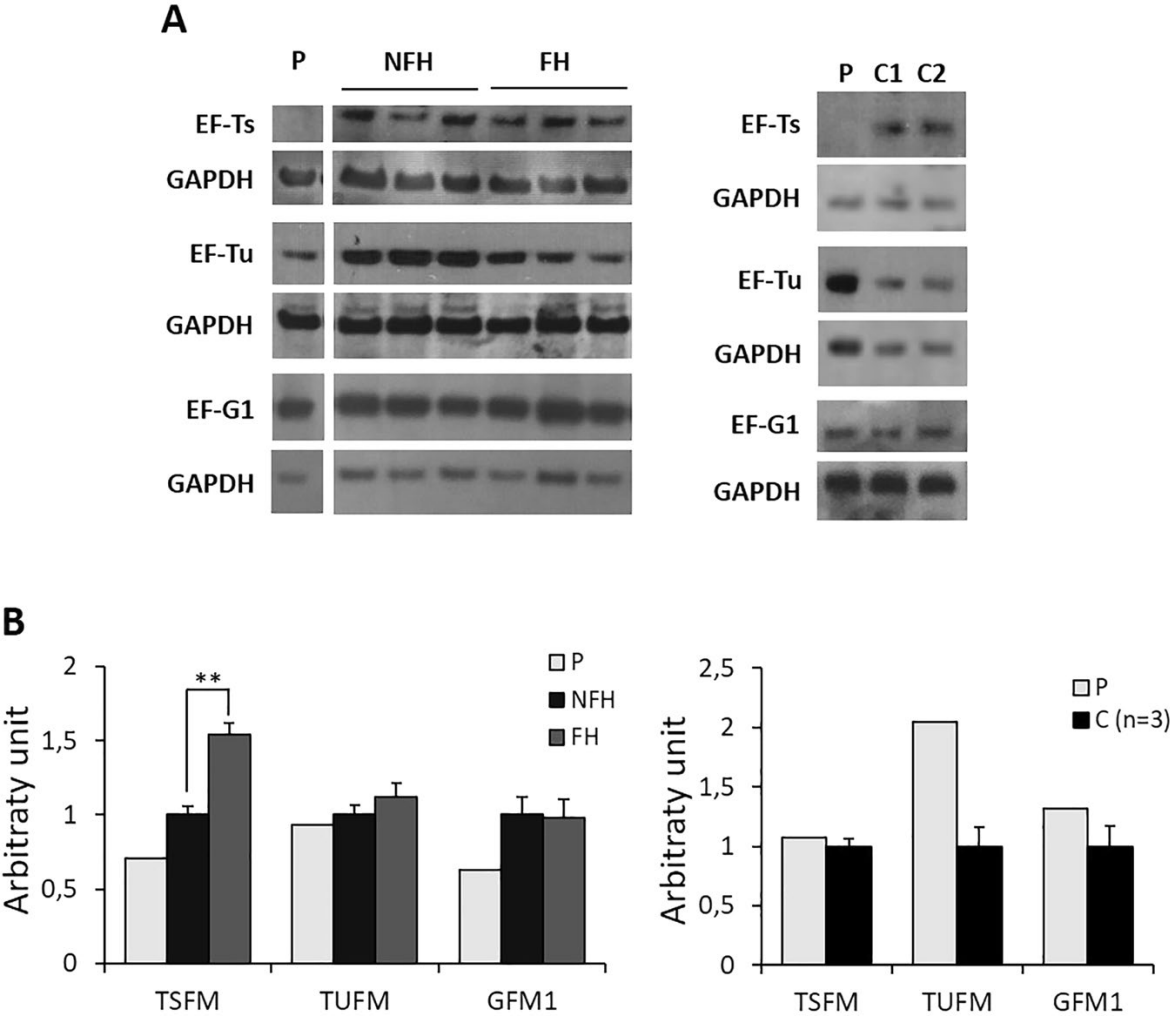
Expression of EF-Ts and EF-Tu on cardiac tissue and fibroblasts. (**A**) Left: Representative western blot images of EF-Ts, EF-Tu and EF-G1 proteins performed on extracts of heart homogenate from patient (P), and controls [i.e. non failing (NFH) and failing heart (FH)]. For each protein the blots are cropped from the same gel. Full-length blots are shown in Supplementary Fig. S5. Right: western blot images of EF-Ts, EF-Tu and EF-G1 proteins performed on fibroblasts derived from patient (P) and controls (i.e. wild-type donors, C). Experiments were performed in triplicate.(**B**) Gene expression analysis of *TSFM*, *TUFM* and *GFM1* performed on cardiac tissue from P, NFH and FH (left), and from patient (P) and controls (C) fibroblasts (right). Data are expressed as mean ± SEM from three separate experiments. **p < 0.*01* for FH versus NFH (umpaired t test).

Both EF-Ts protein and cDNA were dramatically decreased in myocardium from the proband as compared with both FH and NFH, along with a reduction of EF-Tu protein. This last finding is in line with the likely destabilizing effect of the mutations on the EF-Ts/EF-Tu complex, as already reported^18^. In contrast, protein and gene expression of EF-G1 were unchanged. A slight decrease of EF-Tu was also observed in FH *versus* NFH hearts (Fig. 5A,B and Supplementary Fig. S4).

### Induction of mitochondrial biogenesis in mutant fibroblasts associated with decrease of EF-Ts but not EF-Tu proteins

To acquire some clues relating to the tissue-specific presentation of *TSFM* variants, we evaluated the expression of the EF-Ts/EF-Tu complex and the bioenergetic proficiency of skin-derived fibroblasts from the proband and three age-matched adult controls. Interestingly, although EF-Ts levels were decreased in patient fibroblasts, as in cardiac tissue, both EF-Tu protein and gene expression were up-regulated, while EF-G1 was unchanged as compared to controls (n = 3) (Fig. 5A,B and Supplementary Fig. S4).

**Figure 6:**
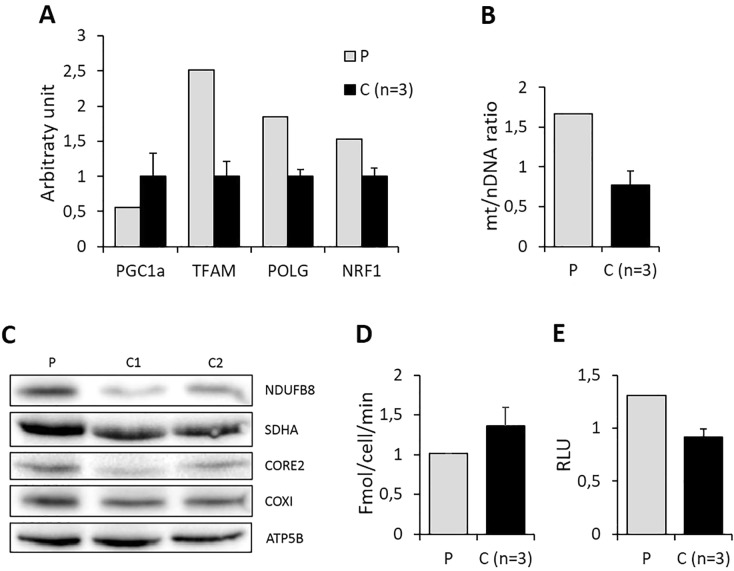
Fibroblasts molecular and biochemical phenotype. (**A**) Expression levels of genes involved in mtDNA transcription and replication in fibroblasts from the proband (P) and controls (C). Data are expressed as mean ± SEM from three separate experiments. (**B**) Amount of mtDNA, expressed as mtDNA/nuclear DNA ratio, in fibroblasts from the proband (P) and controls (C) maintained in galactose medium for 96 hours. Data are expressed as mean ± SEM from three separate experiments. **(C**) Western blot analysis of nuclear- and mitochondrial-encoded proteins on mutant fibroblast and two controls. **(D**) Rate of oxygen consumption in fibroblasts from proband (P) and control (C) incubated for 96 hours in galactose medium. Values obtained in galactose medium are normalized to the value obtained in glucose medium. Data are expressed as mean ± SEM from three separate experiments. (**E**) Levels of hydrogen peroxide in fibroblasts from proband (P) and control (C) incubated for 24 hours in galactose medium. Values obtained in galactose medium are normalized to the value obtained in glucose medium. Data are expressed as mean ± SEM from three separate experiments.

Furthermore, mitochondrial biogenesis appeared to be activated, as demonstrated by the induction of essential genes involved in both mitochondrial transcription and replication (*PGC1α*, *NRF1*, *TFAM* and *POLG*), an increase in mtDNA copy number (Fig. 6A,B) and increased steady-state levels of selected nuclear and mitochondrial-encoded respiratory chain subunits (Fig. 6C). Accordingly, the rate of oxygen consumption, a hallmark of bioenergetic proficiency, was unchanged after 96 hours incubation in glucose-free, galactose-supplemented medium (galactose medium), a condition forcing cells to rely on OXPHOS for ATP production (Fig. 6D). Since a well-known activator of mitochondrial biogenesis is the increase in oxidative stress^21^, we measured the levels of hydrogen peroxide. These were slightly increased in mutant fibroblasts incubated 24 hours in galactose medium, as compared with controls (Fig. 6E).

## Discussion

Herein, we report an unusual presentation of mitochondrial cardiomyopathy, with dilated phenotype and pathologic evidence of biventricular fibro-adipose replacement, in a 33-year-old woman who underwent cardiac transplant. The morphologic evidence of cardiac mitochondrial proliferation and the clinical history of mild ataxia suggested an underlying mitochondrial aetiology, which was confirmed by the demonstration of a combined defect of respiratory chain enzyme activities in the affected myocardium. Analysis of mtDNA was unremarkable, while whole exome sequencing revealed two novel compound heterozygous variants in highly conserved regions of the *TSFM* gene, encoding the mitochondrial translation elongation factor EF-Ts. This protein participates in the elongation step of mitochondrial protein translation by binding and stabilizing the translation elongation factor Tu (EF-Tu) and promoting the formation of EF-Tu/GTP from EF-Tu/GDP^22^. Structural analysis of the homology models built for EF-Ts isoforms indicated a likely destabilizing effect of the mutations on EF-Ts/EF-Tu complex. Accordingly, the steady-state levels of both EF-Ts and EF-Tu proteins in the affected myocardium were dramatically decreased.

Mutations in the *TSFM* gene [i.e. the homozygous p.(Arg312Trp) change, in a subdomain of EF-Ts interacting with EF-Tu], have been previously demonstrated in infantile cases of severe hypertrophic cardiomyopathy with or without encephalomyopathy^18,23^, or liver failure^24^, leading to death in the first months of life. More recently, further *TSFM* pathogenic changes have been demonstrated in children with slowly progressing hypertrophic or dilated cardiomyopathy, who later developed neurological symptoms as the predominant manifestation of the disease (i.e. optic and/or peripheral neuropathy; ataxia; Leigh disease)^19,20^. In the present case, symptoms and sign of cardiac involvement manifested only in adulthood, characterized by fatigue and dyspnoea rapidly progressing to congestive heart failure, while a mild and stable form of ataxia was documented since infancy.

Our report confirms that the heart is a main target of *TSFM* dysfunction, both in children and adults, and extends the morphologic spectrum of mitochondrial cardiomyopathies. In fact, the finding of multiple foci of fibro-adipose myocardial replacement involving both ventricles had never been reported to our knowledge in primary mitochondrial dysfunction. Indeed, this is the pathologic hallmark of ACM, a heritable disease due to genetic defects in cardiac desmosomes, leading to heart failure and lethal arrhythmias, especially in young people and athletes^14^. Although the original phenotype of ACM was characterized by a predominant right ventricle involvement (i.e. right ventricular dysplasia)^25^, clinical variants characterized by equivalent or even predominant involvement of the left ventricle (i.e., biventricular or left-dominant ACM), have been increasingly recognized^26^. Of note, patients with biventricular ACM may manifest clinically only in the advanced disease stage, with heart failure in the absence or presence of ventricular arrhythmias and may be incorrectly diagnosed as having idiopathic DCM^14^. In the present case, mitochondrial aetiology was suggested by the marked mitochondrial proliferation within cardiac myocytes and the anamnestic report of mild neurological manifestations.

Although a full understanding of the pathogenesis of ACM is currently lacking, it is believed that impairment of desmosomal assembly causes the translocation of the desmosomal protein plakoglobin from the sarcolemma to the nucleus, where it may induce a gene transcriptional switch from myogenesis to lipogenesis^14^. Interestingly, studies on induced pluripotent stem cells (iPSCs)-derived cardiomyocytes showed an overall depressed energy metabolism in ACM, characterized by dominant glucose utilization and reduction of fatty acid oxidation, similar to what is usually observed in mitochondrial disease^27^. Accordingly, our findings provide evidence that metabolic derangement may play a role in the development of an “ACM-like phenotype”. It is noteworthy that ACM has been described sporadically in association not only with desmosomal proteins but also with neuromuscular disorders, such as myofibrillar myopathies or myotonic dystrophy^28^.

An interesting finding of our work is related to the different behaviour of EF-Tu expression in the affected myocardium and skin-derived fibroblasts from the proband. In fact, in the myocardium EF-Tu levels were severely reduced, in agreement with previous observations, which led to the suggestion that destabilization of EF-Ts/EF-Tu complex leads to increased turnover of its components and decreased steady-state levels of both factors^18^. In contrast, marked decrease of EF-Ts was associated with an up-regulation of EF-Tu in mutant fibroblasts. Interestingly, previous reports showed that forced overexpression of EF-Tu in cells obtained from carriers of pathogenic *TFAM* mutations is able to rescue the EF-Ts defect^18^. In line with this last observation, bioenergetic proficiency of fibroblasts from the proband appeared within normal limits, even under stressful conditions (i.e. galactose medium), as demonstrated by the oxygen consumption rate. The over-expression of EF-Tu was paralleled by the overall activation of mitochondrial biogenesis, a well-known compensatory phenomenon in mitochondrial disorders^29^, possibly triggered by the increased production of oxygen species^21^. Thus, the induction of an adaptive response involving mitochondrial biogenesis and transcriptional upregulation of EF-Tu is able to reduce the penetrance of the *TSFM* mutations in fibroblasts. This phenomenon does not occur (or is ineffective) in cardiac tissue, in line with previous reports pointing to the impaired mitochondrial biogenesis as a main player in the progression to heart failure^30,31^. In agreement with this suggestion, EF-Tu levels were mildly although significantly reduced also in non-mitochondrial failing hearts.

The ability of increased EF-Tu levels, either due to up-regulation or induced over-expression, to compensate for a reduction in the levels of mutated EF-Ts, is reminiscent of the widely documented ability of several mt-aminoacyl-tRNA synthetases (aaRSs) to “rescue” defects associated with point mutations in cognate and, in some cases, non-cognate, mt-tRNAs (see for a Review^32^). In the case of mutations in mt-tRNAs, as well as EF-Ts, the rescuing effect has been observed both as a consequence of mt-aaRSs up-regulation and induced over-expression^32^. These observations suggest that when a protein involved in mitochondrial protein synthesis is mutated, increasing the levels of one of its interaction partners might be a general mechanism to prevent impairments in this essential cellular process.

In conclusion, our report provides evidence about *TSFM* dysfunction as a cause of mitochondrial cardiomyopathy both in infants and adults, thus extending the array of morphologic phenotypes associated with mitochondrial cardiomyopathies and highlights the role of metabolic derangements in the development of an ACM-like phenotype. Observations on mutant fibroblasts suggest possible explanations for the tissue specific expression of *TSFM*-associated disease, which may be extended to other mitochondrial translation disorders.

## Materials and Methods

### Patients

The work described has been carried out in accordance with The Code of Ethics of the World Medical Association (Declaration of Helsinki) and conformed to Sapienza University of Rome Ethical Committee protocols. All studies were undertaken with written informed patients consent. Collection and experiments with human-derived fibroblast have been approved from Sapienza University of Rome Ethical Committee (Prot. 358/16). Non-failing, myocardial samples (n = 4) were obtained from donors hospitalized at Policlinico Umberto I of Rome, which were unsuitable for transplantation for technical reasons. Both the proband and failing heart (n = 4) were obtained from unrelated patients who underwent cardiac transplantation because of idiopathic DCM at Department of Cardiac Surgery and Transplantation of San Camillo-Forlanini Hospital of Rome. Myocardial samples were referred to the Cardiovascular Pathology Unit of Policlinico Umberto I for diagnostic purposes and examined following our routine diagnostic work-up^1^ and, accordingly to the rules of our local Committee, approval was waived. Skin derived fibroblast’s (n = 3) were obtained from age-matched volunteers.

### Histology, histochemistry and respiratory chain enzyme biochemistry on heart tissue

Immediately after explant, hearts (from proband and controls) were weighed and photographed. Myocardial samples were obtained from multiple sites in both ventricles and were either processed for routine histology, or snap frozen in liquid nitrogen–chilled isopentane for histochemical, biochemical, and molecular studies, according to a standardized protocol^1,33^.

Hematoxylin-eosin and Masson trichrome stains were performed on multiple paraffin sections from both ventricles and the septum. To assess mitochondrial respiratory chain function, sequential COX and SDH reactions on frozen sections from the left and right ventricles were performed as described^1^. For ultrastructural analysis samples were fixed in glutaraldehyde (2.5% in phosphate buffer), postfixed in osmium tetroxide, and embedded in epoxy resin (Epon 812). Ultrathin sections (100 nm) were observed after standard Reynold staining with a Zeiss EM 10 TEM equipped with digital image data processing software (DigitalMicrograph 3.4TM; Gatan, Munchen, Germany).

Biochemical analysis of individual mitochondrial respiratory chain complexes was performed on frozen cardiac muscle homogenates, as described previously^34^. Specific enzyme activities were normalized to that of citrate synthase, a marker of mitochondrial mass.

### Mitochondrial DNA analysis

Total DNA was isolated from heart tissue and fibroblasts using Wizard Genomic DNA Purification Kit (Promega, Madison, WI, USA). The total amount of mtDNA was calculated by quantitative real-time PCR (AB 7500 Fast, Applied Biosystems, Warrington, UK). Briefly, a mtDNA fragment (nt 4625–4714; GenBank reference NC_012920.1) and a nuclear DNA fragment (*FasL* gene) were amplified using a TaqMan-MGB probe system. The amount of mtDNA relative to nuclear genomic DNA was evaluated with the comparative Ct method (2−ΔCt), where ΔCt = Ct mtDNA−Ct nuclear DNA^35^.

The entire mitochondrial genome was amplified and sequenced in a series of overlapping fragments by using BigDye terminator chemistries on an Applied Biosystem 3100 automated sequencer (Applied Biosystems)^35^. All sequences were directly compared with the revised Cambridge reference sequence for human mtDNA (GenBank accession no. NC_012920.1).

By using shifted primer sets within the major arc of human mtDNA, heart tissue DNA was tested for single, large-scale mtDNA deletions as described previously^36^.

### Whole Exome Sequencing

Whole exome sequencing of heart DNA was conducted using the BCM HGSC CORE exome capture design (52 Mb, NimbleGen) and with paired-end sequencing on an Illumina HiSeq. 2500 to a 100X average coverage. Bioinformatic analyses were conducted as previously described^37^. Single nucleotide variants (SNVs) and small insertions and deletions (InDels) were scored by GATK^38^. Quality control filtering of variants was based on coverage, strand bias, mapping quality, and base quality. Custom Perl scripts were used to annotate variants as previously described^37,39^. Algorithms used for prediction of potential functional consequences of variants included CADD^40^, SIFT^41^ and PolyPhen2^42^, Genomic Evolutionary Rate Profiling (GERP)^43^, and PhyloP^44^.

### Cell culture

Fibroblasts were cultured in Dulbecco’s modified Eagle’s medium (DMEM), supplemented with 4.5 g/l D-glucose, 10% fetal bovine serum (FBS), 2 mM L-glutamine, 100 U/ml penicillin and 100 mg/ml streptomycin (referred to as glucose medium) in a humidified atmosphere of 95% air and 5% CO2 at 37 °C. A subset of experiments was performed in parallel either in glucose medium or in glucose free DMEM, supplemented with 10% dialyzed FBS (dFBS), 5 mM galactose and 110 mg/ml sodium pyruvate (referred to as galactose medium).

### Oxygen consumption

Oxygen consumption was measured in treated and untreated intact cells (2–3 × 10^6^) using a Clark-type oxygen electrode (Hansatech, Norfolk, UK) in 1 ml DMEM lacking glucose and supplemented with 10% sodium pyruvate, as described previously^45^.

### Reactive oxygen species evaluation

Levels of hydrogen peroxide (H_2_O_2_) were measured by luminescence assay using a ROS-Glo™ H_2_O_2_ Assay (Promega), according to the manufacturer’s protocol as previously described^46^. Briefly, a number of 1 × 10^4^ cells were plated in a 96-well white cell culture plate and incubated overnight in glucose or galactose medium. After 24 hours medium was supplemented with H_2_O_2_ Substrate Dilution Buffer containing ROS-Glo™ H_2_O_2_. After 2 hours incubation, ROS-Glo™ Detection Solution was added and the plate was incubated for 20 min at room temperature. Luminescence was determined with a GloMax® Multi + Luminometer. The average RLU of triplicate samples were calculated.

### Gene expression analysis by quantitative real-time PCR

Gene expression experiments were performed on cardiac tissue from patient and controls. As controls we used both non-failing heart that were unsuitable for transplantation for technical reasons (NFH, n = 4), and failing heart with DCM obtained from transplant procedure (FH, n = 4). In addition, skin-derived fibroblasts from the proband and three age-matched controls were analyzed. Total RNA was isolated using SV Total RNA isolation kit (Promega) and measured with a NanoDrop ND-1000 spectrophotometer (NanoDrop Technologies, Wilmington, DE, USA). Total RNA (0.1–1 mg) was reverse-transcribed to cDNA using random hexamer primers. The relative expression levels of *TSFM*, *TUFM*, *GFM1*, *PGC1α*, *TFAM*, *POLG and NRF1* were evaluated by using TaqMan probe chemistry and inventoried and custom FAM-labeled TaqMan MGB probes (Applied Biosystems), according to the manufacturer’s instructions. In all samples, the relative expression of each target gene was evaluated as compared with the mean of controls using the comparative threshold cycle (ΔCt) method. All values were normalized for the housekeeper glyceraldehyde-3-phosphate dehydrogenase (*GAPDH*) gene. Each experiment was performed in triplicate.

### Western blot and immunohistochemical analysis

For Western blot analysis, cells were rinsed twice with ice-cold PBS, lysed in ice-cold RIPA buffer [50 mM Tris–HCl pH 8, 150 mM NaCl, 1% NP-40, 0.5% sodium deoxycholate, 1% sodium dodecyl sulfate (SDS), 1 mM phenylmethylsulfonyl fluoride, 10 mg/ml aprotinin, 10 mg/ml leupeptin and 10 mg/ml pepstatin] and centrifuged at 10,000 g for 10 min at 4 °C. Protein concentration was measured by bicinchoninic acid (Beyotime Biotechnology, Haimen, China). Equal amounts of protein (50 mg) were separated by 12% SDS–PAGE and transferred into a nitrocellulose membrane (Millipore, Bedford, MO, USA). Primary antibodies were visualized using horseradish peroxidase-conjugated secondary antibodies (Dako, Glostrup, Denmark). Signals were detected by enhanced chemiluminescence (Amersham Biosciences, UK). The band intensities of western blots were analyzed by using NIH ImageJ (http://rsb.info.nih.gov/ij/).

The following primary antibodies were used: rabbit polyclonal antibody anti-mt EF-Ts (PA5-27652, Thermo Scientific, Rockford USA); rabbit polyclonal antibody anti-mt EF-Tu (PA5-27511, Thermo Scientific, Rockford USA); rabbit polyclonal antibody anti-mt EF-G1 (PA5-57049, Thermo Scientific, Rockford USA); rabbit polyclonal antibody anti-GAPDH (PA1-16777, Thermo Scientific, Rockford USA); mouse monoclonal antibody anti-SDHA (ab14715; Abcam); mouse monoclonal antibody anti-ATP5A (ab110273; Abcam); mouse monoclonal antibody anti-MT-COI (COX1, ab14705; Abcam); mouse monoclonal antibody anti-NDUFB8 (ab110242; Abcam); mouse monoclonal antibody anti-CORE 2 (ubiquinone: cytochrome c reductase core protein 2; ab110252; Abcam); mouse monoclonal antibody anti-Porin (ab14734; Abcam).

### Analysis of *TSFM* gene product sequence and three-dimensional (3D) structure

The sequences of the four protein isoforms encoded by the *TSFM* gene were downloaded from the UniProt Knowledgebase^47^ with UniProt IDs P43897-1, P43897-2, P43897-3 and P43897-4, respectively (corresponding to NCBI IDs: NP_005717.3, NP_001166167.1, NP_001166166.1 and NP_001166168.1, respectively), and aligned to one another (Fig. 3) using the multiple sequence alignment program ClustalO^48^.

The full MSA of members of the EF-Ts protein family (Pfam ID: PF00889) was downloaded from the Pfam database^49^. This alignment encompasses the region 116-274 of EF-Ts isoform 1 and the homologous regions from over 5,000 family members. Sequences that were incomplete at positions 119 and 229, corresponding to 137 and 169 of EF-Ts isoform 1, were eliminated from the downloaded alignment. The MSA thus obtained, comprising over 3,900 sequences from different species, was used to calculate the frequency of occurrence of each residue at positions 119 and 229 (Supplementary Fig. S3).

A molecular model (Fig. 4) was built for each of the four EF-Ts isoforms by homology modelling, using the program SwissModel (https://swissmodel.expasy.org/). The template used for all isoforms was the three-dimensional (3D) structure of *Bos taurus* mt EF-Ts (chain B, residues 56-338) in complex with EF-Tu (chain A, residues 44–452), experimentally determined by X-ray crystallography, which was downloaded from the Protein Data Bank^50^ (PDB ID: 1XB2; Resolution = 2.20 Å). Model visualization and analysis were performed using SwissPDBViewer^51^ and PyMol (http://www.pymol.org/). Residues were defined to be in contact with Leu169 if they have at least one atom at a distance ≤4.5 Å from one atom of Leu169. The EF-Ts surface in contact con EF-Tu was calculated with Naccess^52^.

## Supplementary information

Supplementary Informations: Novel compound mutations in the mitochondrial translation elongation factor (TSFM) gene cause severe cardiomyopathy with myocardial fibro-adipose replacement
