# Supplementary material for: Novel compound mutations in the mitochondrial translation elongation factor (TSFM) gene cause severe cardiomyopathy with myocardial fibro-adipose replacement
Source: Sci Rep. 2019 Mar 25;9:5108. doi: 10.1038/s41598-019-41483-9 (PMC6434145; doi:10.1038/s41598-019-41483-9)
Supplement: Supplementary file 1 — Supplementary Informations: Novel compound mutations in the mitochondrial translation elongation factor (TSFM) gene cause severe cardiomyopathy with myocardial fibro-adipose replacement [file 41598_2019_41483_MOESM1_ESM.pdf]

SUPPLEMENTARY INFORMATIONS

Manuscript title: Novel compound mutations in the mitochondrial translation elongation factor (*TSFM*) gene cause severe cardiomyopathy with myocardial fibro-adipose replacement

Autyhors: Elena Perli, Annalinda Pisano, Ruth I.C. Glasgow, Miriam Carbo, Steven A. Hardy, Gavin Falkous, Langping He, Bruna Cerbelli, Maria Gemma Pignataro, Elisabetta Zacara, Federica Re, Paola Lilla Della Monica, Veronica Morea, Penelope E. Bonnen, Robert W. Taylor, Giulia d'Amati, Carla Giordano

Supplementary Figure S1

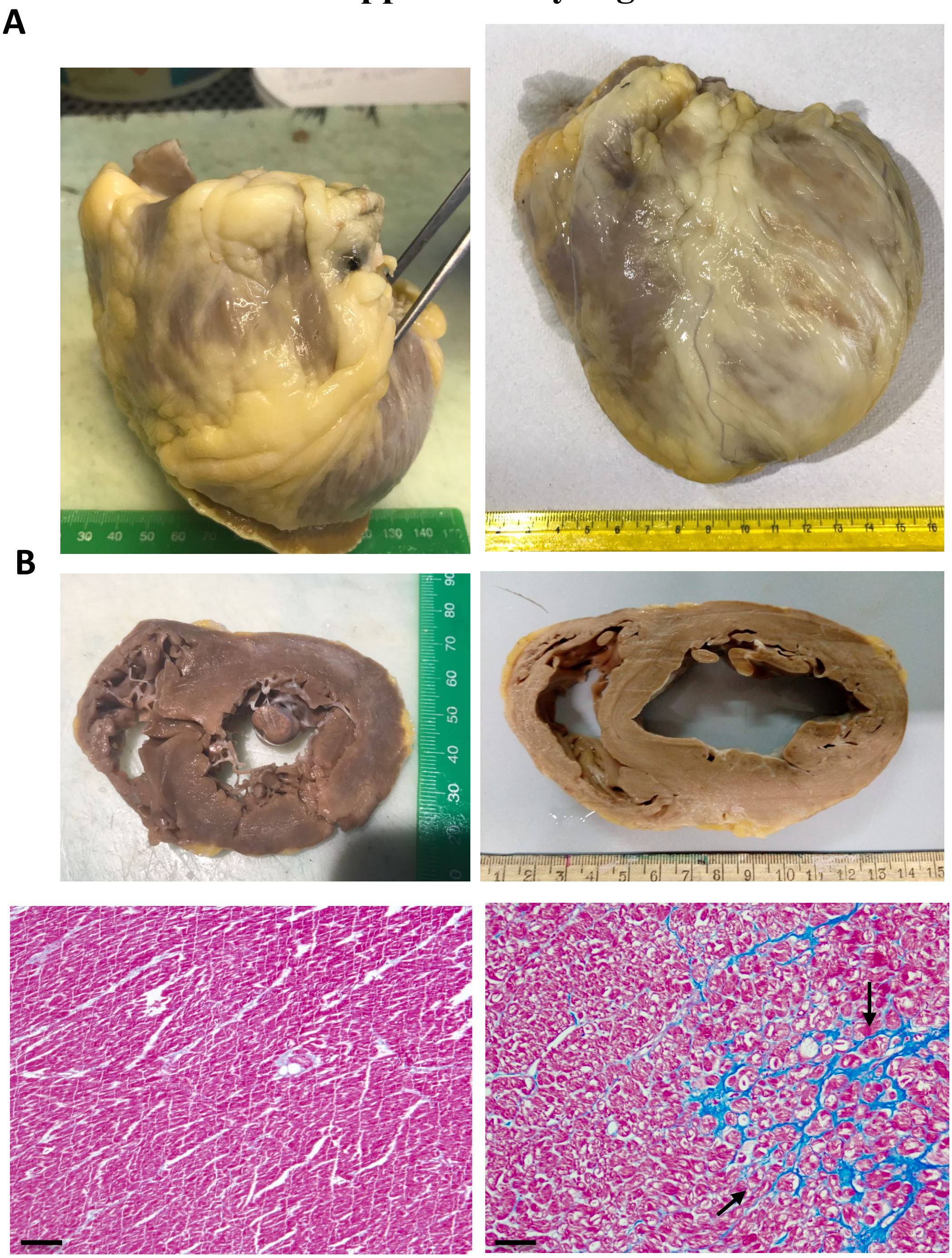

Supplementary Figure S1. Gross and histologic features of non-failing and failing hearts.

A. Frontal view of a non-failing (NFH, left side) and a failing heart (FH, right side) with dilated cardiomyopathy. **B**. Cross section of NFH (left) and FH (right). FH shows biventricular eccentric hypertrophy (i.e. increased mass with biventricular chamber dilation), in absence of myocardial scarring.

C. Histologic picture of the left myocardium highlight the absence of fibrosis in NFH (left side) while FH is characterized by interstitial collagen deposition (in blue, arrows) (Masson trichrome stain, scale bar 250μm)

Supplementary Figure S2

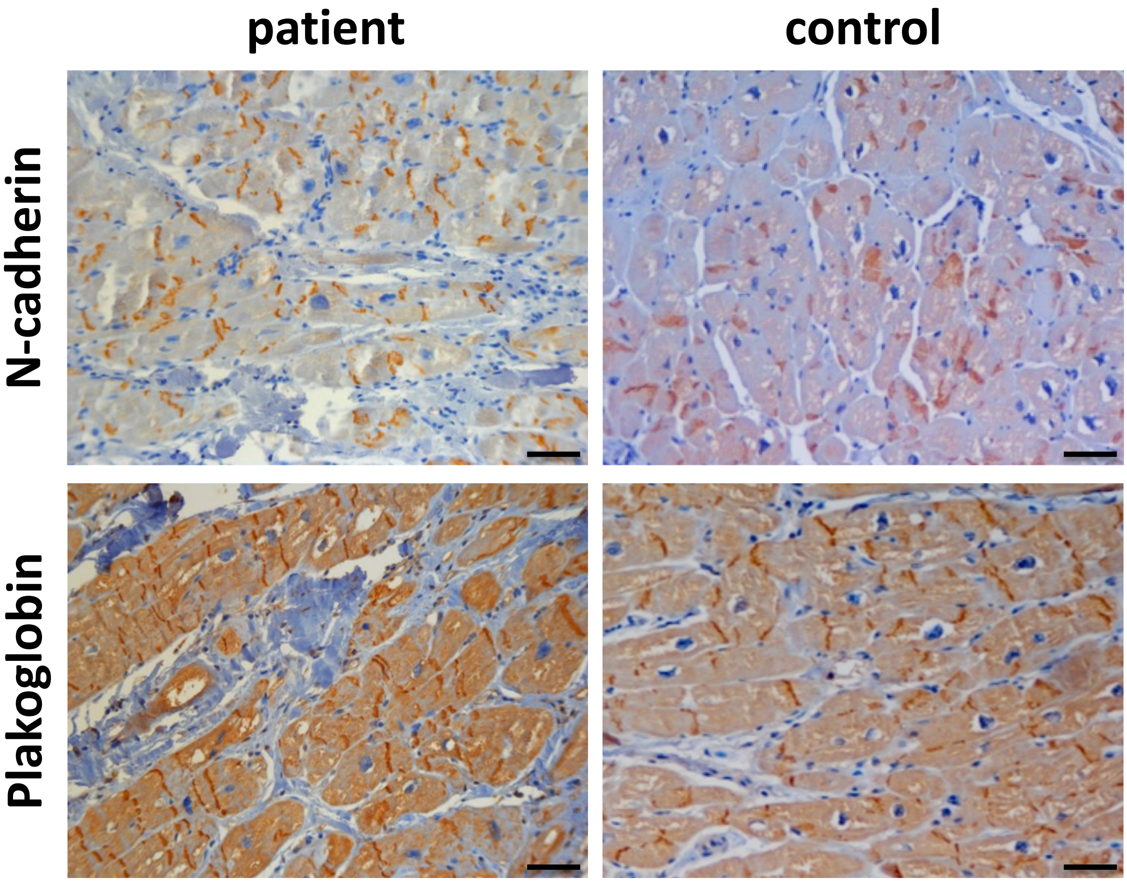

Supplementary Figure S2
Expression of N-cadherin and Plakoglobin on cardiac tissue.
Scale bar 50µm

Supplementary Figure S3

A) Number of occurrences at each alignment position

	ALIGNMENT POSITION																					
	114	115	116	117	118	119	120	121	122	123	124	224	225	226	227	228	229	230	231	232	233	234
A	673	190	0	60	1220	**75**	1616	419	490	207	2300	20	21	27	16	1651	**590**	293	2002	871	296	204
C	0	2	0	9	4	**3**	44	1	3	57	33	2	3	1	0	11	**6**	7	1	62	5	27
D	1225	721	0	2	369	**1**	0	538	531	0	0	19	44	13	64	245	**5**	97	102	8	358	40
E	1358	567	0	31	456	**8**	3	371	729	0	8	30	14	17	38	1064	**4**	91	425	235	479	12
F	1	2	3878	0	4	**1409**	1	1	18	61	3	21	13	2	8	4	**274**	15	13	30	6	322
G	96	310	0	52	263	**2**	210	95	154	3	118	10	8	14	6	64	**28**	119	135	74	14	25
H	5	40	5	24	30	**1**	0	119	20	0	2	0	10	8	2	11	**6**	19	82	21	34	29
I	7	80	5	471	3	**48**	63	7	13	1167	81	6	15	13	22	13	**197**	68	6	134	29	397
K	113	640	0	574	310	**2**	3	616	608	2	9	3	13	44	5	125	**5**	742	232	14	713	10
L	42	126	12	432	11	**1903**	154	64	51	731	592	111	89	36	63	17	**2376**	1431	24	917	10	748
M	3	40	3	32	3	**208**	11	14	28	39	28	6	19	16	7	6	**30**	270	1	67	11	351
N	26	329	0	39	383	**0**	0	675	262	0	2	34	40	33	4	92	**6**	198	217	15	91	33
P	152	59	0	1	0	**0**	0	0	0	0	1	41	23	26	27	59	**9**	17	25	81	690	11
Q	103	351	0	1357	240	**0**	4	355	297	0	9	3	2	3	1	77	**22**	113	147	341	89	12
R	0	90	0	131	65	**0**	0	313	145	0	9	3	4	1	3	12	**11**	45	21	4	28	4
S	86	213	0	30	271	**9**	122	214	291	67	139	37	36	91	78	190	**32**	291	231	399	183	90
T	31	97	2	142	227	**29**	205	109	270	128	298	13	9	22	11	166	**23**	44	116	173	366	184
V	22	87	2	557	30	**145**	1509	15	27	1482	312	8	22	7	27	125	**315**	57	22	198	76	346
Y	1	0	38	1	56	**102**	0	19	7	0	0	4	0	14	8	2	**3**	15	2	16	2	525
W	0	0	0	0	0	**0**	0	0	0	0	0	1	0	0	1	1	**3**	1	2	0	1	119
.	1	1	0	0	0	**0**	0	0	1	1	1	3573	3560	3557	3554	10	**0**	12	139	285	464	456
Total	3945	3945	3945	3945	3945	3945	3945	3945	3945	3945	3945	3945	3945	3945	3945	3945	3945	3945	3945	3945	3945	3945

B) Percentage of occurrences at each alignment position

	ALIGNMENT POSITION																					
	114	115	116	117	118	119	120	121	122	123	124	224	225	226	227	228	229	230	231	232	233	234
A	17	5	0	2	31	**2**	41	11	12	5	58	1	1	1	0	42	**15**	7	51	22	8	5
C	0	0	0	0	0	**0**	1	0	0	1	1	0	0	0	0	0	**0**	0	0	2	0	1
D	31	18	0	0	9	**0**	0	14	13	0	0	0	1	0	2	6	**0**	2	3	0	9	1
E	34	14	0	1	12	**0**	0	9	18	0	0	1	0	0	1	27	**0**	2	11	6	12	0
F	0	0	98	0	0	**36**	0	0	0	2	0	1	0	0	0	0	**7**	0	0	1	0	8
G	2	8	0	1	7	**0**	5	2	4	0	3	0	0	0	0	2	**1**	3	3	2	0	1
H	0	1	0	1	1	**0**	0	3	1	0	0	0	0	0	0	0	**0**	0	2	1	1	1
I	0	2	0	12	0	**1**	2	0	0	30	2	0	0	0	1	0	**5**	2	0	3	1	10
K	3	16	0	15	8	**0**	0	16	15	0	0	0	0	1	0	3	**0**	19	6	0	18	0
L	1	3	0	11	0	**48**	4	2	1	19	15	3	2	1	2	0	**60**	36	1	23	0	19
M	0	1	0	1	0	**5**	0	0	1	1	1	0	0	0	0	0	**1**	7	0	2	0	9
N	1	8	0	1	10	**0**	0	17	7	0	0	1	1	1	0	2	**0**	5	6	0	2	1
P	4	1	0	0	0	**0**	0	0	0	0	0	1	1	1	1	1	**0**	0	1	2	17	0
Q	3	9	0	34	6	**0**	0	9	8	0	0	0	0	0	0	2	**1**	3	4	9	2	0
R	0	2	0	3	2	**0**	0	8	4	0	0	0	0	0	0	0	**0**	1	1	0	1	0
S	2	5	0	1	7	**0**	3	5	7	2	4	1	1	2	2	5	**1**	7	6	10	5	2
T	1	2	0	4	6	**1**	5	3	7	3	8	0	0	1	0	4	**1**	1	3	4	9	5
V	1	2	0	14	1	**4**	38	0	1	38	8	0	1	0	1	3	**8**	1	1	5	2	9
Y	0	0	1	0	1	**3**	0	0	0	0	0	0	0	0	0	0	**0**	0	0	0	0	13
W	0	0	0	0	0	**0**	0	0	0	0	0	0	0	0	0	0	**0**	0	0	0	0	3
.	0	0	0	0	0	**0**	0	0	0	0	0	91	90	90	90	0	**0**	0	4	7	12	12
Total	100	100	100	100	100	**100**	100	100	100	100	100	100	100	100	100	100	**100**	100	100	100	100	100

Supplementary Figure S3. Conservation of leucine residues at positions 137 and 169 of EF-Ts isoform 1. A. Number of occurrence of each amino acid at selected sequence positions in the 3,945 sequences of EF-TS homologues from different species present in the multiple sequence alignment (MSA) analysed in this work (see Materials and Methods). In the left column, amino acids are indicated by one-letter code, and deletions (gaps) by the "." symbol. For clarity, only the alignment regions 114-124 and 224-234, corresponding to 132-142 and 164-174 of EF-TS isoform-1, are shown. Red boxes and font highlight: i) MSA positions 119 and 229, which correspond to 137 and 169 of EF-Ts isoform-1; ii) Amino acids leucine, which is present at positions 137 and 169 of wild-type EF-Ts isoforms, and glycine and phenylalanine, which are present in EF-Ts variants studied in this work, namely p.(Leu137Glyfs*24) and p.(Leu169Phe); iii) the number of sequences having leucine or glycine at position 119, and leucine or phenylalanine at position 229 of the MSA.

B. Frequency of occurrence of each amino acid in the MSA mentioned in panel A. Abbreviations and use of red boxes and font is as in panel A. Cells are colour-coded according to frequency values: 0-9, white; 10-19, pale green; 20-29, green; 30-39, dark green; 40-49, pale yellow; 50-59, yellow; 60-69, dark yellow; 90-99, blue; 100 dark blue.

Supplementary Figure S4

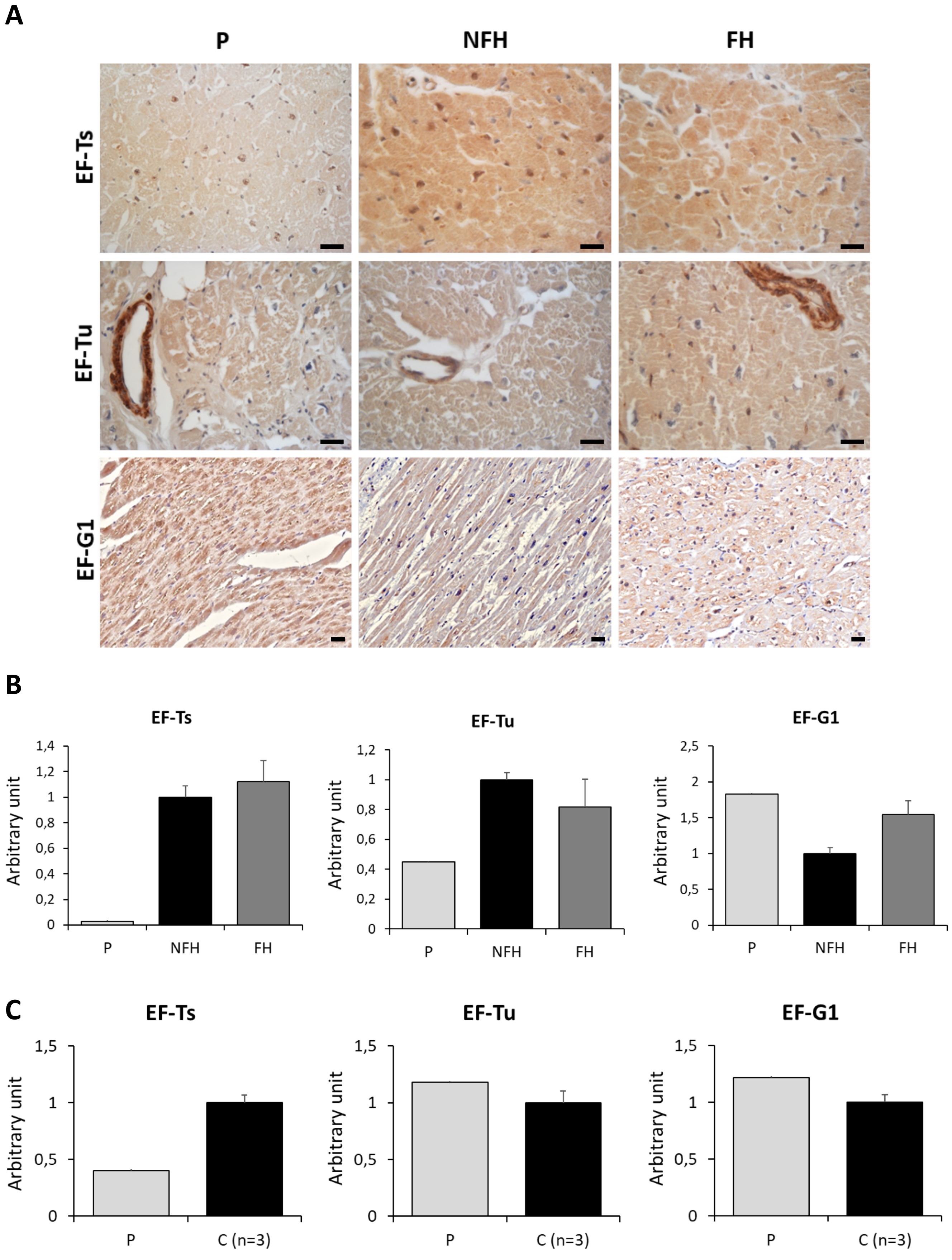

Supplementary Figure S4

Expression of EF-Ts and EF-Tu on cardiac tissue and fibroblasts

A. Representative images of immunostaining for EF-Ts, EF-Tu and EF-G1 antibody in patient (P), non failing (NFH) and failing heart (FH). Scale bar 50µm.

B. and C. Densitometry of Western blot of EF-Ts, EF-Tu and EF-G1 proteins performed on extract of heart homogenate from patient (P), non failing (NFH) and failing heart (FH) **(B)** and fibroblasts derived from and 2 wild-type donors (C) and patient (P) **(C)**. Data are expressed as mean ± S.E.M.

Supplementary Figure S5

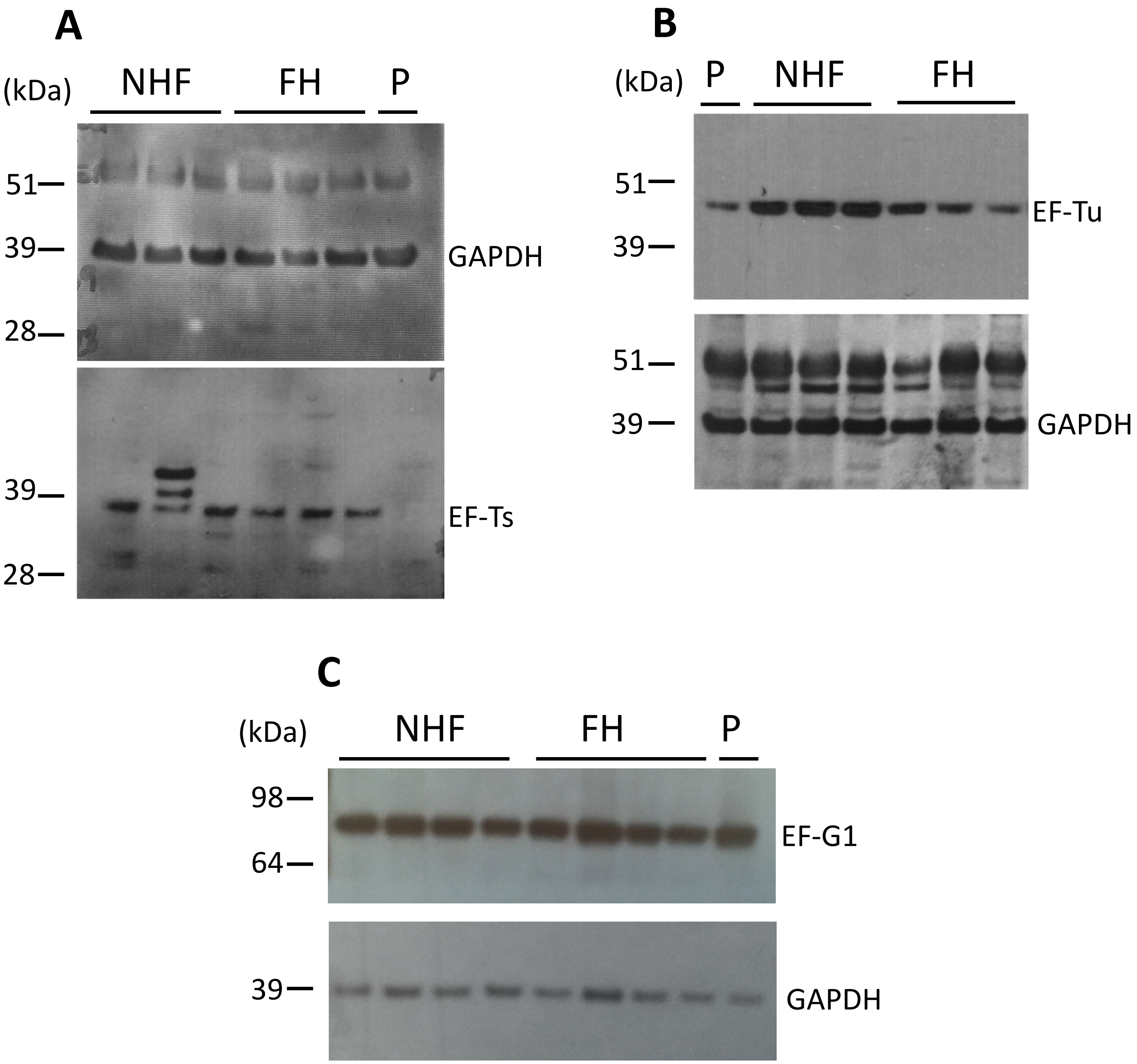

Supplemental Figure S5

Full-length western blot images in Figure 5a for (A) EF-Ts, (B) EF-Tu and (C) EF-G1. GAPDH was used as a loading control for normalization after washing the membrane for 72 hours in PBS.

Supplementary Table S1

Patient	Sex	Age at transplant/ death (years)	Clinical diagnosis	Heart weight (g)	Chamber diameter (mm)	
					LV	RV
IC	F	33	iDCM	360	55	50
FH1	F	58	iDCM	410	54	40
FH2	M	35	iDCM	340	50	40
FH3	F	26	iDCM	370	56	49
FH4	F	42	iDCM	350	70	32
NFH1	F	21	DH	320	20	30
NFH2	M	39	DH	390	30	30
NFH3	F	29	DH	300	20	30
NFH4	F	34	DH	270	20	25

Supplementary Table S1
Demographic data and hearts measures of the index case (IC) and controls. FH=failing heart, NFH=non-failing heart, LV=left ventricle, RV=right ventricle, M=male, F=female, iDCM= idiopathic dilated cardiomyopathy, DH= donor heart

Measures refer to formalin-fixed hearts.

Supplementary Table S2

mtDNA polymorphisms	Protein variations
m.72T>C	Non-coding
m.264G>C	Non-coding
m.309insC	Non-coding
m.750A>G (MTRNR1)	Non-coding
m.1438A>G (MTRNR1)	Non-coding
m.2581A>G (MTRNR2)	Non-coding
m.3766T>C (MTND1)	syn
m.4769A>G (MTND2)	syn
m.5315A>G (MTND2)	syn
m.8668T>C (MTATP6)	p.(Trp48Arg)
m.8860A>G (MTATP6)	p.(Thr112Ala)
m.11440G>A (MTND4)	syn
m.15326A>G (MTCYB)	p.(Thr194Ala)
m.16093T>C	Non-coding
m.16519T>C	Non-coding

Supplementary Table S2
Sequence variations in mtDNA from the proband relative to the revised Cambridge reference sequence (GenBank accession no NC_012920.1). All the identified changes correspond to previously reported polimorphisms listed in MitoMAP compendium (www.mitopat.org, Jun 28th 2018).
